# Advanced bone age in the affected side predicts worse radiographic progression in patients with juvenile idiopathic arthritis and unilateral wrist disease

**DOI:** 10.1186/1546-0096-12-S1-P18

**Published:** 2014-09-17

**Authors:** Giulia Camilla Varnier, Nicola Ullman, Alessandro Consolaro, Roberto Gastaldi, Natascia Di iorgi, Mohamad Maghnie, Cecilia Bava, Alberto Martini, Angelo Ravelli

**Affiliations:** 1 IRCCS G.Gaslini, Genova, Italy

## Introduction

Previous anecdotal observations have suggested that patients with juvenile idiopathic arthritis (JIA) and unilateral wrist synovitis often have an advanced bone age in the affected side and that advancement in skeletal maturation may be associated with development of long-term structural joint damage. However, it is unclear whether the risk of damage in the affected wrist is greater than that of JIA patients with bilateral wrist disease.

## Objectives

To compare the amount of radiographic damage in the wrists between patients with unilateral and bilateral wrist synovitis.

## Methods

21 patients with unilateral wrist synovitis and 21 patients with bilateral wrist synovitis who underwent longitudinal bilateral hand/wrist radiographs were evaluated. Bone age in each wrist was assessed on radiograph made at first observation by two experienced pediatric endocrinologists according to Greulich & Pyle atlas. Endocrinologist assessments were then averaged and the time lag between chronological age and bone age was calculated. Radiographic damage was assessed at baseline and last follow-up visit by measuring carpo-metacarpal ratio (Poznanski score). The Poznanski score was evaluated separately in patients with unilateral wrist disease, whereas in patients with bilateral wrist disease the score of the two wrists was averaged. The demographic and clinical features, including disease duration at first and last radiographic assessments, were comparable between patients with unilateral and bilateral wrist disease. However, patients with unilateral wrist disease had more frequently oligoarthritis (52.3%), whereas patients with bilateral wrist disease had more frequently systemic arthritis (52.3%).

## Results

Bone age was advanced by > 6 months in the affected side in 12/21 patients (57.1%) with unilateral wrist disease and in the right and left side in 8/21 (38.1%) and 7/21 (33.3%) patients with bilateral wrist disease. Comparison of chronological-bone age lag and radiographic damage on baseline and follow-up films in patients with unilateral and bilateral wrist disease is shown in table 1.

**Table 1:** 

	Bone-chronological age lag (years)	Baseline Poznanski score	Follow-up Poznanski score
Unilateral-affected side	1.03	-1.94^£^	-3.95^§^

Unilateral-unaffected side	0.63	-0.47^£^	-1.68^§^

Bilateral-average L/R wrist	0.23	-0.83^$^	-2.74

^$^P=0.03; ^£^p=0.002; ^§^p=0.02

## Conclusion

Our results show that JIA patients with unilateral wrist disease often have advanced skeletal maturation in the affected side and that this is associated by a greater destructive course. This indicates that these patients deserve a careful radiographic follow-up and an early aggressive therapy aimed at suppressing joint inflammation in the wrist to prevent progression of joint damage.

## Disclosure of interest

None declared.

